# Assessment of a Non-Randomized Education Intervention for Primary School Aimed to Promote the Inclusion of People with Celiac Disease: Zeliakide Project (Part II)

**DOI:** 10.3390/nu18111798

**Published:** 2026-06-03

**Authors:** Maialen Vázquez-Polo, Virginia Navarro, Arrate Lasa, Idoia Larretxi, Gesala Perez-Junkera, Silvia Matias, Edurne Simón, Itziar Churruca

**Affiliations:** 1 Gluten 3S Research Group, Department of Nutrition and Food Science, Faculty of Pharmacy, University of the Basque Country (UPV/EHU), 01006 Vitoria-Gasteiz, Spain; maialen.vazquez@ehu.eus (M.V.-P.); arrate.lasa@ehu.eus (A.L.); idoia.larrechi@ehu.eus (I.L.); gesala.perez@ehu.eus (G.P.-J.); silvia.matias@ehu.eus (S.M.); edurne.simon@ehu.eus (E.S.); itziar.txurruka@ehu.eus (I.C.); 2 Nutrition and Food Safety Group, Bioaraba, 01009 Vitoria-Gasteiz, Spain

**Keywords:** nutrition education, celiac disease, gluten-free diet, children, social inclusion, school

## Abstract

Background and Aim: The gluten-free diet (GFD) can have a huge impact on the quality of life of people with celiac disease (CD), especially on a social level. The objective of this work is to evaluate a structured nutrition education program focused on CD and GFD that aims to increase knowledge and improve inclusion attitudes about the disease in children. Methods: This is a one-month intervention for school children aged 10–12 years called Zeliakide (8 sessions). It was carried out through a STEAM methodology, using inquiry-based learning. The participants responses were evaluated through questionnaires before and after the intervention, and participants were also followed up one month later. The control group was a similar group of students who followed their regular school curriculum. Results: 299 children from one school of Vitoria-Gasteiz took part in the study (155 intervention group; 144 control group). Zeliakide significantly improved knowledge about CD and GFD in children, and this knowledge was retained for one month. Concretely, students increased their ability to explain what CD is, to assess gluten, and to classify food groups according to gluten content. The intervention contributed to augmenting the selection of behaviors to overcome differences between individuals, assessed one month after the intervention. In addition, the program allowed students to understand the work of scientists. Conclusions: Zeliakide can contribute to nutrition education initiatives that aim to improve knowledge of CD and GFD in the general population, while promoting empathetic behavior towards people with CD. Registration: clinicaltrials.gov, NCT05467865 on 21 July 2022.

## 1. Introduction

Celiac disease (CD) is a systemic condition where the immune system is activated by the ingestion of gluten and related prolamins in genetically predisposed individuals [1,2]. Symptoms are varied and unspecific, and are mainly gastrointestinal, such as diarrhea, chronic constipation, bloating and abdominal pain, although there has been a shift toward increased recognition of extraintestinal manifestations [1]. These include iron-deficiency anemia, osteoporosis, and less common effects like infertility, migraines, or neurologic and psychiatric symptoms [3]. About 1–2% of the population are diagnosed with CD, although this varies by country and region, and a significant percentage of people remain undiagnosed [3,4,5,6,7].

The only treatment for CD is currently a strict lifelong gluten-free diet (GFD). Gluten is found in various cereals such as wheat, rye or barley, and some oats, and in the many foods made from these cereals, so it is imperative to avoid foods containing them. In order for GFD to be safe, it is necessary to take precautions to prevent cross-contact, which makes adoption of the diet even more complicated [8,9,10,11].

In addition to the importance of the safety of the GFD, as it is a restrictive diet, it is important to consider the nutritional deficiencies that it may generate [12,13,14]. Previous studies have found that the GFD is deficient in complex carbohydrates, dietary fibre, and some vitamins and minerals, and is also high in sugar, saturated fats, and proteins [13,15,16]. The GFD must be balanced, as must the diet of the general population, in order to achieve an optimal state of health [17]. However, specific gluten-free products have a poorer nutritional quality than their gluten-containing analogues and therefore it is difficult to achieve a balanced diet in persons affected with CD [18,19,20,21].

Moreover, dietary restriction can have a huge impact on the quality of life of people with CD, especially on a social level [22,23,24,25,26]. Eating out, attending events, etc. is complicated as finding gluten-free dishes and making sure that they have not been cross-contacted is very difficult [24,27,28,29]. In addition, these difficulties can result in psychological complications, for example, feeling different or excluded [30,31,32,33,34]. Specifically, in a study conducted in Spain, it was found that the greatest concern of Spanish children with CD aged 8–18 years was their relationship with their friends [35]. Furthermore, the disease not only has an impact on the individuals who suffer from it, but also on the people around them. This is because the illness impacts day-to-day tasks, social engagements, and living expenses (given the higher cost of gluten-free products), along with affecting the well-being of caregivers, leading to heightened levels of anxiety [36,37,38,39,40,41].

Several authors have reported that health education should be developed and provided to people with CD in order to help them cope with their situation [42,43,44]. In recent years, several interventions have been designed for the treatment and prevention of other diseases with a high prevalence, but few studies have addressed the celiac community [45,46,47,48]. Moreover, these few studies tend to focus on the patient and not on the general population, despite the fact that the latter plays an important role in the quality of life of those affected by CD. In fact, it has become evident that the general population has little knowledge about CD, and concern has been raised about how this reality may influence patients [49,50,51,52,53,54,55]. It is believed that increasing the awareness and knowledge of CD in society can contribute to improving the social burden of having CD [50,53]. As mentioned, this is of special importance for children.

Many nutrition education programs focus on the school environment, in order to effectively promote health habits from an early age [46,47,48,56], but such programs are not usually focused on CD. New methodological approaches such as STEAM (Science, Technology, Engineering, Arts, and Mathematics) are now being introduced in schools and these approaches are starting to be introduced in nutrition education programs [57,58].

In view of the above, it seems important to develop innovative nutritional education interventions on CD and GFD for children in order to improve their knowledge and attitudes to promote the inclusion of people with CD in society and to encourage development of their scientific skills. Therefore, this trial was designed to evaluate the effectiveness of the Zeliakide program, a one-month STEAM-based nutrition education intervention that specifically focuses on CD and GFD in school children aged 10–12 years.

## 2. Materials and Methods

The program comprised eight sessions of approximately three hours each, delivered over a four-week period. All the children in the 5th and 6th grade were recruited. All children within the same school, academic year, and class group should be included in the intervention, as ethically it is important that all groups have access to the same learning opportunities during the school year. Therefore, the trial was non-randomized and included a control group for comparison in the 6th grade. The participants were evaluated before and after the intervention, and they were also followed up one month later. Parents and guardians were also involved in the intervention and were able to contribute their point of view. The recruitment period occurred between October and December 2020. The intervention was conducted from mid-January to mid-February 2021.

The selected teaching approach was an Inquiry-Based Learning (IBL) method within the STEAM framework. This methodology emphasizes interactive activities and experiments to facilitate learning [59].

The program was carefully designed from a pedagogical point of view and focused on eight distinct competencies and their corresponding learning outcomes. The first three competencies are related to a balanced diet and the remaining five competences to CD and the GFD. To understand the complexity of CD and its treatment, the general population must first understand what a balanced diet is, as it is the basis of the gluten-free diet. The positive results of the first part of the Zeliakide program that focuses on a balanced diet have been previously published [60], as well as the study protocol of the full program (registered in clinicaltrials.gov, NCT05467865 on 21 July 2022) [61]. A more detailed description of the whole program is available in Supplementary Materials File S1.

The present article specifically examines the last five competencies of the study protocol (C4–C8) and their associated learning outcomes, which pertain to the concepts of CD and the GFD and are classified according to Bloom’s taxonomy (Table 1). The taxonomy has been used to classify learning outcomes according to their level of abstraction (the more abstract, the more complex, on a 1 to 6 scale) [62,63]. This article aims to investigate the effectiveness of Zeliakide in enhancing specific knowledge about CD and the GFD, as well as improving related attitudes. At the same time, it also seeks to encourage children’s interest in science.

### 2.1. Participants

The research was conducted with children aged 10 to 12 years who were in the last two years of one primary school in Vitoria-Gasteiz. In order to be eligible for participation in the study, the children had to attend a primary school that implemented project-based learning and to have expressed an interest in being part of the project. Additionally, the schools attended needed to have at least one class in either the fifth or sixth grade. The required number of students necessary to detect a small to medium effect size was 144, which was determined and published in a previous paper [61].

Before the study began, all participants provided informed consent. This included the schools, as well as the teachers, and the parents or guardians of the children involved, who signed consent forms. The research was approved by the Ethics Committee for Human Research at the University of the Basque Country, UPV/EHU (reference number M10/2020/081, 1 July 2020).

### 2.2. Activities Description

To develop the competences and achieve the learning outcomes outlined in Table 1, the students engaged in five activities, organized in small groups of 4 to 5 subjects. These activities were guided by members of the research group, with support from the schoolteachers.

Puzzle game to learn the characteristics of the disease: students were given colored stickers containing information about the characteristics of CD. Each sticker color covered a sub-topic, such as the symptoms of the condition, what the diet should be like for people with CD, and how they felt. Those holding stickers of the same color were asked to form a group and discuss what was written on their stickers. Next, each group shared the information on their stickers aloud. The person leading the session distributed the stickers containing information from other groups to those who did not yet have them. They had to stick these stickers onto a worksheet, thereby completing the puzzle that provided all the information about the characteristics of CD.

Case study to work on inclusion, discussion: The children were presented with a situation where they had to discuss among themselves how they should act. The situation was as follows: “a friend is coming to your house for lunch and she/he has CD”. In addition, they were to come up with proposals to make the person with CD be more included. All the children’s answers were collected and shared on a virtual platform.

Experiment 1: Children analyzed the elasticity and touch/texture of different gluten and gluten-free doughs made with different types of flour (wheat, rice and corn). They extracted the gluten from the doughs.

Experiment 2: Bread and gluten-free bread were sensorially analyzed. The children looked at differences in appearance, texture and taste, smell, and touch.

Experiment 3: They developed an immunochromatography experiment to detect the presence or absence of gluten in different food samples.

Experiment 4: They studied the labeling of different packaged foods to see whether or not they contained gluten, and how the presence or absence of gluten was signaled.

It should be noted that the children’s parents were involved in the activities through exercises they carried out at home in relation to what was done in the class-sessions: they reflected about the difficulties faced by people with CD, they repeated experiment 2 with another food, and they repeated experiment 4 in the supermarket.

### 2.3. Data Collection

To evaluate the efficacy of the program, participants were requested to fill out a questionnaire prior to the intervention, as well as after its completion (pre and post questionnaires, respectively), in the classroom. Moreover, a third questionnaire was administered one month post-intervention to determine whether the acquired knowledge had been retained by the participants (RT-retention questionnaire). The questionnaires were meticulously crafted in close collaboration with educators and had undergone evaluation in previous pilot studies. This process facilitated the incorporation of substantial improvements, especially with regard to the adaptations to the public’s cognitive capacity (Supplementary Materials File S2). Parents or guardians completed another questionnaire to assess the children’s interest from their perspective and to gauge their own opinion.

### 2.4. Control Group

The control group consisted of a similar number of students to the intervention group, from the same school, and with similar socio-demographic traits, even though the students were in their last year of the academic cycle. They followed their regular school curriculum without any additional intervention. Like the intervention group, students in the control group were asked to complete a questionnaire when the post questionnaire of the intervention was administered for the other group.

### 2.5. Statistical Analysis

All the answers to the open-ended questions were categorized [64]. One judge created the categories for organizing the responses, while two different judges independently assigned the responses to the established categories. When there was a high level of agreement between the two assessing judges (K-value above 0.7), the categorization of the responses was deemed accurate. In cases where the two evaluators had differing classifications for an answer, a consensus was reached after a face-to-face discussion. The categories ranked the responses on a scale from 0 to X (the maximum value depended on the categories determined by the judges). The scale ranged from 0 for the least accurate responses to X for the most accurate responses. Responses were sometimes reported as “student knows the answer” or “student does not know the answer” when the categorization scale ranged from 0 to 1. The responses subjected to categorization are clearly identified in the tables. The judges were part of the research team.

The statistical analysis was conducted using the IBM SPSS Statistics for Windows, version 28.0 software (IBM Corp., Armonk, NY, USA). Descriptive analysis was performed on the socio-demographic variables and response variables. The data distribution was found to be non-normal. To compare the pre, post, and RT questionnaires, Wilcoxon tests were used for quantitative responses, the McNemar test for dichotomous qualitative variables, and Friedman and Marginal Homogeneity tests for qualitative variables with more than two response options. The Mann--Whitney U test was used to compare the responses of boys and girls. A significant difference between the pre, post, and RT questionnaires was determined at *p* < 0.05.

For comparing the intervention group with the control group, the Mann--Whitney U test was employed for quantitative variables, and the Chi-square test for qualitative variables.

## 3. Results

### 3.1. Sample Characteristics

A total of 310 children from 12 classrooms were eligible for the study, and all agreed to participate and signed the informed consent. Of these, 155 participants (6 classrooms) were assigned to the intervention group and 155 (6 classrooms) to the control group. All participants in the intervention group completed the follow-up, whereas 11 participants in the control group did not. In total, 299 participants were included in the analysis, with 155 assigned to the intervention group and 144 assigned to the control group. Among the participants, there were 147 girls, 149 boys, and 3 individuals who identified themselves with another gender.

In the intervention group, the mean age was 10.1 ± 0.3, while in the control group, it was 11.5 ± 0.5. The intervention group comprised children in the 5th grade of primary school, whereas the control group consisted of children in the 6th grade. All the children in the study came from a comparable socio-economic background and they belonged to the same school in Vitoria-Gasteiz city.

Among the participants, 1 (0.3%) person had CD, 6 people (2.1%) had a cohabiting family member with CD, 91 (30.4%) people had a family member or friend with CD, 105 (35.1%) had an acquaintance with CD, 82 (27.4%) did not know anyone with CD, and 14 people (4.7%) did not respond to the question.

### 3.2. Outcomes by Competence

#### 3.2.1. C4: Explaining CD to Others

In order to evaluate this learning outcome, four questions were included in the questionnaires. The results are shown in Table 2. It is noticeable that participants were able to provide better explanations about the condition of CD after the intervention.

#### 3.2.2. C5: Assessing the Impact of Own Actions on Others—Empathetic Behavior

To assess these learning outcomes, three questions were formulated. As can be seen in Table 3, the number of children who demonstrate empathetic behavior and select behaviors to overcome differences between people is higher after the intervention.

#### 3.2.3. C6: Analyzing Gluten

To assess these learning outcomes, participants were asked one open question: “Where is the gluten?”. The results showed the participants were clearly more aware of where gluten is after the intervention, with correct answers increasing from 1.79 ± 1.79 to 2.91 ± 1.52, with 4 the maximum (*p* < 0.001). Furthermore, one more question was added to the post questionnaire: “What makes a dough elastic?”. This question was not included in the pre questionnaire as it was incomprehensible to the children before talking about the topic. After the intervention, the majority of the participants knew that gluten gives elasticity to the dough, with almost 80% of the responses correct. Detailed results can be found in Table S3.1 of the Supplementary Materials File S3.

#### 3.2.4. C7: Classifying Food Groups According to Gluten Content

In order to evaluate these learning outcomes, children were asked four questions. The responses are shown in Table 4. Following the intervention, there was a significant improvement in students’ ability to categorize foods based on their gluten content. An additional question was included in the post questionnaire, which was not present in the pre questionnaire because it was too complex for the children to understand before discussing the topic. This final response revealed that following the intervention over half of the participants knew how to identify the “gluten-free” symbols.

#### 3.2.5. C8: Understanding the Work of Scientists: Analyzing Gluten Content in Foods Through Experimentation

All children actively participated in the proposed experiments and had a chance to analyze and evaluate the results obtained. In this case, four statements were proposed in the questionnaire for them to indicate their degree of agreement. The detailed results are shown in Table S3.2 from Supplementary Materials File S3.

After the intervention, there was an increase in consensus among the students regarding the necessity of conducting laboratory experiments to detect gluten. They also expressed a stronger belief in the importance of scientists conducting experiments and showed a statistically significantly greater interest in pursuing a career in science.

Both boys and girls increased their interest in becoming scientists in the future, based on their responses to the third statement (‘In the future, I would like to become a scientist’). However, although they started from a similar pre status, the boys’ change in interest was more pronounced (the difference between post and pre scores in boys was 0.39 ± 0.93 and in girls 0.15 ± 1.09, with the difference by sex between −0.09 and 0.58, with a confidence interval of 95% and a *p*-value of 0.08). Among the answers to the fourth statement (‘Researchers are crazy, weird and male’), more girls than boys (21 vs. 9) selected the minimum score in the pre, while in the post the difference was smoothed out.

### 3.3. Results After 1 Month

To assess the long-term maintenance of competences, some of the questions from the pre and post questionnaires were included in the RT questionnaire. The specific questions and corresponding answers included in the RT questionnaire are presented in Table 5.

The results indicate that, for the majority of questions, knowledge levels one month after the intervention were higher than before the intervention. Furthermore, with respect to selecting empathetic behaviors, there was an increase in the number of children choosing empathic behaviors one month after the intervention than before the intervention.

In the question “Where is the gluten?”, a slight decrease in knowledge was observed between the post and the RT questionnaire. However, for several other questions, an increase in knowledge was observed from the post to the RT questionnaire.

### 3.4. Control Group Results

A selection of the most significant questions was made to compare the intervention group with the control group. The detailed results are presented in Table S3.3 in the Supplementary Materials File S3. Briefly, initial knowledge is generally similar in both groups (when comparing pre data with control data). Exceptionally, the control group seems to identify gluten as responsible for CD better than the experimental group before the intervention. However, in the rest of the items it did not score better, so the location of gluten in the diet, the symptoms it produces, and the importance of science in detecting it was similar in the control group and in the experimental group before the intervention. As was expected, the control group knew less than the intervention group at post and RT, and, in addition, they chose less empathetic attitudes.

### 3.5. Parents Reported Results

The questionnaire answered by the participants’ parents collected information about the children’s interest in Zeliakide and their own opinion of it. The results showed that the vast majority of participants talked about the program at home and more than 70% reported they had taught their parents something they did not know. Both children and parent’s interest in the program was notable. Most of the participants carried out the activities that were proposed to be done at home. A total of 93.5% of parents would recommend the Zeliakide program to other schools. Detailed results are shown in Tables S3.4 and S3.5 in the Supplementary Materials File S3.

## 4. Discussion

To the authors’ knowledge, Zeliakide is the first nutrition education program focused on CD that targets the general population. Several educational interventions on CD have been carried out, but most of them focused only on people with CD and sought to improve dietary adherence and/or reduce symptoms [65,66,67,68,69,70,71]. Furthermore, there are also many studies on the influence of CD on the quality of life and daily life of people with CD [10,22,23,24,25,26,32]. However, not much effort has been made to address the general population.

Zeliakide was shown to be effective in raising knowledge about CD and the GFD and supporting its retention by children, as can be seen in the competences C4, C6, and C7. The learning outcomes of these competences are at levels 2 (understand), 3 (apply), and 4 (analyze) of Bloom’s taxonomy, which means that the children are able to achieve advanced cognitive skills that will empower them to deal with complex situations and demonstrate empathy towards their peers. It is noticeable that, while the control group represented the best choice available, disparities were observable in the pre questionnaire responses of the experimental group. In some instances, the control group exhibited a modest level of knowledge regarding experiments and labels. However, in other instances, the control group demonstrated a higher level of knowledge, including the identification of gluten as the causative agent for the observed symptoms and the identification of the food group in which gluten is present. The underlying causes of this discrepancy remain unclear, as it is challenging to prevent the dissemination of information both within and outside the school. Nevertheless, the results demonstrated a substantial shift in knowledge after the intervention, which is a noteworthy and substantial outcome.

Moreover, during the Zeliakide program, students were able to understand the work of scientists. They carried out experimental work and observed, analyzed, and evaluated the results (C8), applying the STEAM approach of the program. This approach can enable the provision of individuals with the necessary skills and knowledge to drive innovation, boost productivity, enhance global competitiveness, and contribute to social progress in fields such as health and education. Moreover, after the intervention, the participants showed that they knew the importance of scientists carrying out experiments in the laboratory, and their interest in science increased. In contrast, the control group did not attach as much importance or show as much interest.

This more complex competence has been achieved probably in part thanks to the Inquiry-Based Learning (IBL) methodology used where the competences are acquired through the ideas that arise through experimentation, as has been reported in several studies [59,72,73,74].

On the other hand, the intervention was able to promote empathetic behaviour towards people with CD and increase the selection of behaviours to overcome differences between individuals. These changes were maintained one month after the intervention (C5). It was observed that the control group chose less empathetic behaviors. As mentioned before, it is believed that achieving a society that is both aware of CD and empathetic would be one of the key ways to improve the social problems faced by people with CD [43,75]. Moreover, it is anticipated that as a result of the intervention, participants would become more empathetic to others who may be different in some respects. The results obtained are consistent with those of Russo et al., who studied the impact of CD in children on their families. They found that the (non-celiac) siblings of children with CD felt that they had developed more empathy for others by experiencing their siblings’ disease at close quarters [76]. In another study by Oflu et al., it was found that children with CD were also more empathetic than healthy children [77]. So, it could be said that understanding what CD is, in a detailed and informed way, can contribute to increased empathy towards the people around us.

It is interesting to note that policy-makers and practitioners are becoming increasingly worried about the lack of representation of girls and women in STEAM education [78]. In fact, many efforts are currently being made to increase girls’ interest in science [79,80,81,82]. While Zeliakide was not specifically focused on girl’s engagement, it was carried out mainly by women, who act as a reflection of women’s presence in science for the girls in the classroom. Nevertheless, while the results showed that girls had a less stereotypical view of scientists, they did not visualize themselves as easily as boys in being scientists in the future. Even so, their interest was high after the intervention. Probably, more effort should be made to reinforce the presence of women in this kind of intervention throughout the entire academic life of girls.

For the Zeliakide program to be effective, family involvement has been essential. Family members participated in the activities to be carried out at home by the children. Almost all the children’s parents reported that the children had talked about the activities carried out in class and the majority stated that they had learned something new about the subject from their children. In addition, the parents thought that Zeliakide was very interesting. This is an important point as several studies have highlighted the importance of involving the family in children’s nutritional education programs, either in person [83,84,85] or through homework [86,87,88]. Moreover, beyond the participants’ learning, the parents reported that their interest in the activity was high.

One limitation of the study is that it was conducted in just one school and city. Moreover, the control group only filled out the questionnaire once to reduce time consumption and minimize disruption to the children’s educational routine; moreover, they were one year older. Despite this, both the intervention group and the control group studied nutrition-related topics as part of their regular school curriculum, with the exception of the Zeliakide program, which was exclusive to the intervention group. It is important to emphasize that the utilization of a control group for the purpose of comparison is not a customary element in such interventions, thereby augmenting the study’s robustness. Nevertheless, the aforementioned factors give rise to the potential for bias.

It is also of note that Zeliakide was primarily designed for young children, who were at a crucial stage of cognitive development and that it aligns with the school curriculum, building upon existing knowledge to foster new learning. It is notable for its focus on the school context, emphasizing pedagogical learning through assessment of competencies and outcomes, strategically implemented at a time when students had acquired the required skills. It is worth noting the innovative social approach of the intervention, emphasizing inclusion and overall social well-being, which shifts attention from the individual patient to the broader community.

## 5. Conclusions

The Zeliakide program effectively increased knowledge and improved attitudes toward CD among children aged 10–12 years. Therefore, Zeliakide contributes to the need for structured nutrition education initiatives grounded in pedagogical principles in this area, which are essential to support the social inclusion of individuals with CD.

## Figures and Tables

**Table 1:** Learning outcomes, activities, and outcome measures regarding the last five competences of the Zeliakide program (C4 to C8).

Competence	Learning Outcome	Activity	Outcomes
C4. The student should be able to explain what celiac disease is.	*Bloom level 2. Understanding* The student acknowledges that gluten harms people with CD and makes them sick. The student concludes that people with CD should follow a gluten-free diet.	Game to learn the characteristics of the disease (Puzzle).	Change in Celiac Disease knowledge.
C5. The student should be able to assess the impact of their own actions on others.	*Bloom level 6. Evaluation* The student demonstrates empathetic behavior. The student selects actions and behaviors to overcome differences between people.	Game to learn the characteristics of the disease (Puzzle). Case study to work on inclusion: discussion.	Change in intentional behavior related to the social inclusion of people with CD: perception of the influence of one’s own actions on others.
C6. The student should be able to analyze gluten.	*Bloom level 3. Application* The student manipulates gluten and identifies it. The student figures out that gluten gives elasticity to doughs.	Experiment 1: Learning by doing: dough made with different types of flour. Experiment 2: Food analysis through the senses.	Change in knowledge regarding gluten and its presence in foods.
C7. The student should be able to classify food groups according to gluten content.	*Bloom level 4. Analysis* The student identifies the food groups that contain gluten: placing them in cereals. The student concludes that processed foods may contain gluten. The student deduces where gluten is by analyzing food labels.	Experiment 3: Gluten detection experiment, immunochromatography. Experiment 4: Study of food labeling.	
C8. The student should be able to understand the work of scientists, to know that the gluten content of foods is analyzed through experimentation.	*Bloom level 6. Evaluation* The student is able to perform experimental work, observe, analyze, and evaluate the results.	All the experiments.	Change in attitude in relation to interest in science.

*Bloom level*: Bloom’s degree of cognitive abstraction [62,63].

**Table 2:** Questions and answers to evaluate competence 4: explaining CD to others.

Question	Type of Response	Pre Response Quantity (%) or Mean ± SD	Post Response Quantity (%) or Mean ± SD	*p*
How much do you know about CD?	Scale 0 (nothing)–4 (much)	1.99 ± 1.17	2.85 ± 1.06	<0.001
What symptoms do people with CD have? (*open question*)	He/She/They know the answer	15 (9.7%)	93 (60%)	<0.001
	He/She/They do not know the answer	133 (85.8%)	51 (32.9%)	
	No response	7 (4.5%)	11 (7.1%)	
When do people with CD develop symptoms? (*open question*)	Scale 0 (do not know the answer)–3 (do know the answer)	0.57 ± 1.11	1.06 ± 1.28	<0.001
What compound in food is harmful to people with CD? (*open question*)	He/She/They know the answer	33 (21.3%)	82 (52.9%)	<0.001
	He/She/They do not know the answer	114 (73.5%)	63 (40.6%)	
	No response	8 (5.2%)	10 (6.5%)	

CD: celiac disease; SD: standard deviation.

**Table 3:** Questions and answers to evaluate competence 5: assessing the impact of own actions on others.

Question	Type of Response	Pre Response Quantity (%) or Mean ± SD	Post Response Quantity (%) or Mean ± SD	*p*
How do you think people with CD feel? (*open question*)	He/She/They wonder how they feel	86 (55.5%)	116 (74.8%)	<0.001
	He/She/They do not wonder how they feel	61 (39.4%)	29 (18.7%)	
	No response	8 (5.1%)	10 (6.5%)	
Does a person with CD enjoy eating?	Scale 0 (nothing)–4 (much)	2.16 ± 1.03	2.24 ± 1.11	NS
What would you do if it was your birthday and a classmate had CD?	Bring the cake that I like the most	6 (3.9%)	4 (2.6%)	<0.001
	Bring the cake that I like the most and another gluten-free cake for the person with CD	62 (40%)	21 (13.5%)	
	Bring a gluten-free cake for everyone	82 (52.9%)	124 (80%)	
	No response	5 (3.2%)	6 (3.9%)	

CD: celiac disease; NS: non-significant; SD: standard deviation.

**Table 4:** Questions and answers to evaluate the competence 7: classifying food groups according to gluten content.

Question	Type of Response	Pre Response Quantity (%) or Mean ± SD	Post Response Quantity (%) or Mean ± SD	*p*
Which of the following foods a person with CD cannot eat?	He/She/They know the answer	70 (45.2%)	102 (65.8%)	<0.001
	He/She/They do not know the answer	82 (52.9%)	45 (29%)	
	No response	3 (1.9%)	8 (5.2%)	
From the following list of foods, which ones may contain gluten?	Score 0–11 (number of correct answers)	6.40 ± 2.75	8.39 ± 2.66	<0.001
Can a prepared food (cream of vegetables, prepared beans…) have gluten, even if it is made from gluten-free ingredients?	He/She/They know the answer	102 (65.8%)	122 (78.7%)	<0.001
	He/She/They do not know the answer	45 (29%)	26 (16.8%)	
	No response	8 (5.2%)	7 (4.5%)	
How can we know (without experimenting) whether a food contains gluten or not? (*open question*)	Scale between 0 (do not know the answer)–3 (do know the answer)	0.59 ± 1.09	2.21 ± 1.30	<0.001
Which symbols indicate that a food does not contain gluten? (*open question*)	He/She/They know the answer	-	100 (64.5%)	-
	He/She/They do not know the answer	-	48 (31.0%)	-
	No response	-	7 (4.5%)	-

CD: celiac disease; SD: standard deviation.

**Table 5:** Results obtained from RT questionnaire.

Question	Type of Response	Pre Response Quantity (%) or Mean ± SD	Post Response Quantity (%) or Mean ± SD	RT Response Quantity (%) or Mean ± SD	*p* (Pre-RT)	*p* (Post-RT)
How much do you know about CD?	Scale 0 (nothing)–4 (much)	1.99 ± 1.17	2.85 ± 1.06	3.12 ± 0.78	<0.001	<0.05
What symptoms do people with CD have? (*open question*)	He/She/They know the answer	15 (9.7%)	93 (60%)	106 (68.4%)	<0.001	0.087
	He/She/They do not know the answer	133 (85.8%)	51 (32.9%)	40 (25.8%)		
	No response	7 (4.5%)	11 (7.1%)	9 (5.8%)		
When do people with CD develop symptoms? (*open question*)	Scale 0 (do not know the answer)–3 (do know the answer)	0.57 ± 1.11	1.06 ± 1.28	1.74 ± 1.44	<0.001	<0.001
What compound in food is harmful to people with CD? (*open question*)	He/She/They know the answer	33 (21.3%)	82 (52.9%)	86 (55.5%)	<0.001	NS
	He/She/They do not know the answer	114 (73.5%)	63 (40.6%)	59 (38.1%)		
	No response	8 (5.2%)	10 (6.5%)	10 (6.5%)		
What would you do if it was your birthday and a classmate had CD?	Bring the cake that I like the most	6 (3.9%)	4 (2.6%)	9 (5.8%)	<0.05	<0.05
	Bring the cake that I like the most and another gluten-free cake for the person with CD	62 (40%)	21 (13.5%)	32 (20.6%)		
	Bring a gluten-free cake for everyone	82 (52.9%)	124 (80%)	105 (67.7%)		
	No response	5 (3.2%)	6 (3.9%)	9 (5.8%)		
Where is the gluten? (*open question*)	Scale between 0 (do not know the answer)–4 (do know the answer)	1.79 ± 1.79	2.91± 1.52	2.48 ± 1.67	<0.01	<0.01
Can a prepared food (cream of vegetables, prepared beans…) have gluten, even if it is made from gluten-free ingredients?	He/She/They know the answer	102 (65.8%)	122 (78.7%)	113 (72.9%)	NS	NS
	He/She/They do not know the answer	45 (29%)	26 (16.8%)	33 (21.3%)		
	No response	8 (5.2%)	7 (4.5%)	9 (5.8%)		
How can we know (without experimenting) whether a food contains gluten or not? (*open question*)	Scale between 0 (do not know the answer)–3 (do know the answer)	0.59 ± 1.09	2.21 ± 1.30	2.07 ± 1.33	<0.001	NS

CD: celiac disease; NS: non-significant; SD: standard deviation.

## Data Availability

The original contributions presented in this study are included in the article/Supplementary Materials. Further inquiries can be directed to the corresponding author.

## Supplementary Materials

The following supporting information can be downloaded at: https://www.mdpi.com/article/10.3390/nu18111798/s1, File S1. Key aspects of Zeliakide program; File S2: Questionnaire to evaluate the program; File S3: Detailed results tables.

## Abbreviations

The following abbreviations are used in this manuscript:

GDF	Gluten-free Diet
CD	Celiac Disease
IBL	Inquiry-Based Learning
STEAM	Science, Technology, Engineering, Arts, and Mathematics

## References

[1] Husby S., Koletzko S., Korponay-Szabó I.R., Mearin M.L., Phillips A., Shamir R., Troncone R., Giersiepen K., Branski D., Catassi C. (2012). European Society for Pediatric Gastroenterology, Hepatology, and Nutrition Guidelines for the Diagnosis of Coeliac Disease. J. Pediatr. Gastroenterol. Nutr..

[2] Husby S., Koletzko S., Korponay-Szabó I., Kurppa K., Mearin M.L., Ribes-Koninckx C., Shamir R., Troncone R., Auricchio R., Castillejo G. (2020). European Society Paediatric Gastroenterology, Hepatology and Nutrition Guidelines for Diagnosing Coeliac Disease 2020. J. Pediatr. Gastroenterol. Nutr..

[3] Lebwohl B., Rubio-Tapia A. (2021). Epidemiology, Presentation, and Diagnosis of Celiac Disease. Gastroenterology.

[4] King J.A., Jeong J., Underwood F.E., Quan J., Panaccione N., Windsor J.W., Coward S., deBruyn J., Ronksley P.E., Shaheen A.A. (2020). Incidence of Celiac Disease Is Increasing Over Time: A Systematic Review and Meta-Analysis. Am. J. Gastroenterol..

[5] Stahl M., Li Q., Lynch K., Koletzko S., Mehta P., Gragert L., Norris J.M., Andrén Aronsson C., Lindfors K., Kurppa K. (2023). Incidence of Pediatric Celiac Disease Varies by Region. Am. J. Gastroenterol..

[6] Singh P., Arora A., Strand T.A., Leffler D.A., Catassi C., Green P.H., Kelly C.P., Ahuja V., Makharia G.K. (2018). Global Prevalence of Celiac Disease: Systematic Review and Meta-Analysis. Clin. Gastroenterol. Hepatol..

[7] Catassi C., Gatti S., Fasano A. (2014). The New Epidemiology of Celiac Disease. J. Pediatr. Gastroenterol. Nutr..

[8] Aljada B., Zohni A., El-Matary W. (2021). The Gluten-Free Diet for Celiac Disease and Beyond. Nutrients.

[9] Mulder C.J., van Wanrooij R.L., Bakker S.F., Wierdsma N., Bouma G. (2013). Gluten-Free Diet in Gluten-Related Disorders. Dig. Dis..

[10] Bascuñán K.A., Vespa M.C., Araya M. (2017). Celiac Disease: Understanding the Gluten-Free Diet. Eur. J. Nutr..

[11] Francavilla R., Cristofori F., Stella M., Borrelli G., Naspi G., Castellaneta S. (2014). Treatment of Celiac Disease: From Gluten-Free Diet to Novel Therapies. Minerva Pediatr..

[12] Jamieson J.A., Neufeld A. (2020). Food Sources of Energy and Nutrients among Canadian Adults Following a Gluten-Free Diet. PeerJ.

[13] Cardo A., Churruca I., Lasa A., Navarro V., Vázquez-Polo M., Perez-Junkera G., Larretxi I. (2021). Nutritional Imbalances in Adult Celiac Patients Following a Gluten-Free Diet. Nutrients.

[14] Vici G., Belli L., Biondi M., Polzonetti V. (2016). Gluten Free Diet and Nutrient Deficiencies: A Review. Clin. Nutr..

[15] Churruca I., Miranda J., Lasa A., Bustamante M., Larretxi I., Simon E. (2015). Analysis of Body Composition and Food Habits of Spanish Celiac Women. Nutrients.

[16] González T., Larretxi I., Vitoria J.C., Castaño L., Simón E., Churruca I., Navarro V., Lasa A. (2018). Celiac Male’s Gluten-Free Diet Profile: Comparison to That of the Control Population and Celiac Women. Nutrients.

[17] Penagini F., Dilillo D., Meneghin F., Mameli C., Fabiano V., Zuccotti G. (2013). V Gluten-Free Diet in Children: An Approach to a Nutritionally Adequate and Balanced Diet. Nutrients.

[18] Miranda J., Lasa A., Bustamante M.A., Churruca I., Simon E. (2014). Nutritional Differences Between a Gluten-Free Diet and a Diet Containing Equivalent Products with Gluten. Plant Foods Hum. Nutr..

[19] Segura M.E., Rosell C.M. (2011). Chemical Composition and Starch Digestibility of Different Gluten-Free Breads. Plant Foods Hum. Nutr..

[20] Larretxi I., Simon E., Benjumea L., Miranda J., Bustamante M.A., Lasa A., Eizaguirre F.J., Churruca I. (2019). Gluten-Free-Rendered Products Contribute to Imbalanced Diets in Children and Adolescents with Celiac Disease. Eur. J. Nutr..

[21] Fry L., Madden A.M., Fallaize R. (2018). An Investigation into the Nutritional Composition and Cost of Gluten-Free versus Regular Food Products in the UK. J. Hum. Nutr. Diet..

[22] Sevinç E., Çetin F.H., Coşkun B.D. (2017). Psychopathology, Quality of Life, and Related Factors in Children with Celiac Disease. J. Pediatr. (Rio. J).

[23] Simsek S., Baysoy G., Gencoglan S., Uluca U. (2015). Effects of Gluten-Free Diet on Quality of Life and Depression in Children with Celiac Disease. J. Pediatr. Gastroenterol. Nutr..

[24] Zysk W., Głąbska D., Guzek D. (2018). Social and Emotional Fears and Worries Influencing the Quality of Life of Female Celiac Disease Patients Following a Gluten-Free Diet. Nutrients.

[25] Meyer S., Monachesi C., Barchetti M., Lionetti E., Catassi C. (2023). Cross-Cultural Participation in Food-Related Activities and Quality of Life among Children with Celiac Disease. Children.

[26] Bouery P., Attieh R., Sacca L., Sacre Y. (2024). Assessment of the Social Quality of Life and the Physical Activity of Adult Celiac Disease Patients Following a Gluten-Free Diet in Lebanon. Nutr. Health.

[27] Parsons K., Brown L., Clark H., Allen E., McCammon E., Clark G., Oblad R., Kenealey J. (2021). Gluten Cross-Contact from Common Food Practices and Preparations. Clin. Nutr..

[28] Falcomer A.L., Santos Araújo L., Farage P., Santos Monteiro J., Yoshio Nakano E., Puppin Zandonadi R. (2020). Gluten Contamination in Food Services and Industry: A Systematic Review. Crit. Rev. Food Sci. Nutr..

[29] Guennouni M., Admou B., El Khoudri N., Bourrhouat A., Zogaam L.G., Elmoumou L., Hilali A. (2022). Gluten Contamination in Labelled Gluten-Free, Naturally Gluten-Free and Meals in Food Services in Low-, Middle- and High-Income Countries: A Systematic Review and Meta-Analysis. Br. J. Nutr..

[30] Rosén A., Ivarsson A., Nordyke K., Karlsson E., Carlsson A., Danielsson L., Högberg L., Emmelin M. (2011). Balancing Health Benefits and Social Sacrifices: A Qualitative Study of How Screening-Detected Celiac Disease Impacts Adolescents’ Quality of Life. BMC Pediatr..

[31] Olsson C., Lyon P., Hörnell A., Ivarsson A., Sydner Y.M. (2009). Food That Makes You Different: The Stigma Experienced by Adolescents With Celiac Disease. Qual. Health Res..

[32] Cadenhead J.W., Wolf R.L., Lebwohl B., Lee A.R., Zybert P., Reilly N.R., Schebendach J., Satherley R., Green P.H.R. (2019). Diminished Quality of Life among Adolescents with Coeliac Disease Using Maladaptive Eating Behaviours to Manage a Gluten-Free Diet: A Cross-Sectional, Mixed-Methods Study. J. Hum. Nutr. Diet..

[33] Wolf R.L., Lebwohl B., Lee A.R., Zybert P., Reilly N.R., Cadenhead J., Amengual C., Green P.H.R. (2018). Hypervigilance to a Gluten-Free Diet and Decreased Quality of Life in Teenagers and Adults with Celiac Disease. Dig. Dis. Sci..

[34] Singh P., Dennis M. (2018). Eliminating Dietary Gluten: Don’t Be a “Glutton for Punishment”. Dig. Dis. Sci..

[35] Barrio J., Cilleruelo M.L., Román E., Fernández C. (2018). Health-Related Quality of Life in Spanish Coeliac Children Using the Generic KIDSCREEN-52 Questionnaire. Eur. J. Pediatr..

[36] Germone M.M., Ariefdjohan M., Stahl M., Shull M., Mehta P., Nagle S., Tarbell S., Liu E. (2022). Family Ties: The Impact of Celiac Disease on Children and Caregivers. Qual. Life Res..

[37] Satherley R.M., Coburn S.S., Germone M. (2020). The Impact of Celiac Disease on Caregivers’ Well-Being: An Integrative Review. J. Pediatr. Gastroenterol. Nutr..

[38] Abreu Paiva L.M., Gandolfi L., Pratesi R., Harumi Uenishi R., Puppin Zandonadi R., Nakano E.Y., Pratesi C.B. (2019). Measuring Quality of Life in Parents or Caregivers of Children and Adolescents with Celiac Disease: Development and Content Validation of the Questionnaire. Nutrients.

[39] Malik R. (2019). The Significant Psychological Burden of a Celiac Family: Overlooked and Untreated. Indian J. Pediatr..

[40] Ludvigsson J.F., Roy A., Lebwohl B., Green P.H., Emilsson L. (2017). Anxiety and Depression in Caregivers of Individuals with Celiac Disease—A Population-Based Study. Dig. Liver Dis..

[41] Lee A.R., Wolf R.L., Lebwohl B., Ciaccio E.J., Green P.H.R. (2019). Persistent Economic Burden of the Gluten Free Diet. Nutrients.

[42] Casellas F., Burgos R., Marcos A., Santos J., Ciriza-de-los-Ríos C., García-Manzanares Á., Polanco I., Puy-Portillo M., Villarino A., Lema-Marqués B. (2018). Documento de Consenso Sobre las Dietas de Exclusión en El Síndrome Del Intestino Irritable (SII). Rev. Española Enfermedades Dig..

[43] Langarizadeh M., Rahmati P., Yousefpour Azari S., Sarpourian F., Sayadi M.J., Langarizadeh M.H., Fatemi Aghda S.A. (2023). Identifying and Validating the Educational Needs to Develop a Celiac Self-Care System. BMC Prim. Care.

[44] Samasca G., Sur G., Lupan I., Deleanu D. (2014). Gluten-Free Diet and Quality of Life in Celiac Disease. Gastroenterol. Hepatol. Bed Bench.

[45] Li B., Liu W.J., Adab P., Pallan M., Hemming K., Frew E., Lin R., Martin J., Liu W., Cheng K.K. (2017). Cluster-Randomised Controlled Trial to Assess the Effectiveness and Cost-Effectiveness of an Obesity Prevention Programme for Chinese Primary School-Aged Children: The CHIRPY DRAGON Study Protocol. BMJ Open.

[46] Patriota P.F., Filgueiras A.R., de Almeida V.B.P., Alexmovitz G.A.C., da Silva C.E., de Carvalho V.F.F., Carvalho N., de Albuquerque M.P., Domene S.M.A., do Prado W.L. (2017). Effectiveness of a 16-Month Multi-Component and Environmental School-Based Intervention for Recovery of Poor Income Overweight/Obese Children and Adolescents: Study Protocol of the Health Multipliers Program. BMC Public Health.

[47] Li B., Pallan M., Liu W.J., Hemming K., Frew E., Lin R., Liu W., Martin J., Zanganeh M., Hurley K. (2019). The CHIRPY DRAGON Intervention in Preventing Obesity in Chinese Primary-School--Aged Children: A Cluster-Randomised Controlled Trial. PLoS Med..

[48] Xu H., Li Y., Zhang Q., Hu X.L., Liu A., Du S., Li T., Guo H., Li Y., Xu G. (2017). Comprehensive School-Based Intervention to Control Overweight and Obesity in China: A Cluster Randomized Controlled Trial. Asia Pac. J. Clin. Nutr..

[49] Zipser R.D., Farid M., Baisch D., Patel B., Patel D. (2005). Physician Awareness of Celiac Disease: A Need for Further Education. J. Gen. Intern. Med..

[50] Aziz I., Karajeh M.A., Zilkha J., Tubman E., Fowles C., Sanders D.S. (2014). Change in Awareness of Gluten-Related Disorders among Chefs and the General Public in the UK: A 10-Year Follow-up Study. Eur. J. Gastroenterol. Hepatol..

[51] Karajeh M.A., Hurlstone D.P., Patel T.M., Sanders D.S. (2005). Chefs’ Knowledge of Coeliac Disease (Compared to the Public): A Questionnaire Survey from the United Kingdom. Clin. Nutr..

[52] Simpson S., Lebwohl B., Lewis S.K., Tennyson C.A., Sanders D.S., Green P.H. (2011). Awareness of Gluten-Related Disorders: A Survey of the General Public, Chefs and Patients. E-SPEN Eur. E-J. Clin. Nutr. Metab..

[53] Taskin B., Savlak N. (2021). Public Awareness, Knowledge and Sensitivity towards Celiac Disease and Gluten-Free Diet Is Insufficient: A Survey from Turkey. Food Sci. Technol..

[54] Qasem W.A., Roumi A.A., Al Mojil K., Sakijha H., AlMughamis N. (2023). Awareness of Celiac Disease among the Public in Kuwait: A Cross-Sectional Survey. BMC Res. Notes.

[55] Alhussain M.H. (2021). Awareness of Celiac Disease among the General Public in Saudi Arabia. Int. J. Celiac Dis..

[56] Minossi V., Pellanda L.C. (2015). The “Happy Heart” Educational Program for Changes in Health Habits in Children and Their Families: Protocol for a Randomized Clinical Trial. BMC Pediatr..

[57] Bayles J., Peterson A.D., Jilcott Pitts S., Bian H., Goodell L.S., Burkholder S., Hegde A.V., Stage V.C. (2021). Food-Based Science, Technology, Engineering, Arts, and Mathematics (STEAM) Learning Activities May Reduce Decline in Preschoolers’ Skin Carotenoid Status. J. Nutr. Educ. Behav..

[58] Bucher Della Torre S., Akré C., Suris J.C. (2010). Obesity Prevention Opinions of School Stakeholders: A Qualitative Study. J. Sch. Health.

[59] Arriassecq I., Greca I., Cayul E. (2017). Secuencias de Enseñanza y Aprendizaje Basadas En Resultados de Investigación: Propuesta de un Marco Teórico Para El Abordaje de la Teoría Especial de la Relatividad. Enseñanza Cienc. Rev. Investig. Exp. Didácticas.

[60] Vázquez-Polo M., Churruca I., Lasa A., Perez-Junkera G., Larretxi I., Bustamante M.Á., Martinez O., Navarro V. (2026). Effectiveness of a Controlled Trial of Nutrition Education in Primary School, ZELIAKIDE Project: Knowledge and Mid-Morning Snack Consumption. BMC Public Health.

[61] Vázquez-Polo M., Churruca I., Perez-Junkera G., Larretxi I., Lasa A., Esparta J., Cantero-Ruiz de Eguino L., Navarro V. (2024). Study Protocol for a Controlled Trial of Nutrition Education Intervention about Celiac Disease in Primary School: ZELIAKIDE Project. Nutrients.

[62] Bloom B.S., Krathwohl D.R. (1956). Taxonomy of Educational Objetives: The Classification of Educational Goals, by a Committee of College and University Examiners. Handbook I: Cognitive Domain.

[63] Anderson L. (2014). Taxonomy of Educational Objetives.

[64] Stokes P., Urquhart C. (2013). Qualitative Interpretative Categorisation for Efficient Data Analysis in a Mixed Methods Information Behaviour Study. Inf. Res..

[65] Akbari Namvar Z., Mahdavi R., Shirmohammadi M., Nikniaz Z. (2022). The Effect of Group-Based Education on Gastrointestinal Symptoms and Quality of Life in Patients with Celiac Disease: Randomized Controlled Clinical Trial. BMC Gastroenterol..

[66] Suárez-González M., Bousoño-García C., Jiménez-Treviño S., Díaz-Martín J.J. (2021). Gluten-Free Diet: Nutritional Strategies to Improve Eating Habits in Children with Celiac Disease: A Prospective, Single-Arm Intervention Study. Nutrients.

[67] Nikniaz Z., Namvar Z.A., Shirmohammadi M., Maserat E. (2022). Smartphone Application for Celiac Patients: Assessing Its Effect on Gastrointestinal Symptoms in a Randomized Controlled Clinical Trial. Int. J. Telemed. Appl..

[68] Dowd A.J., Kronlund L., Warbeck C., Parmar C., Daun J.T., Wytsma-Fisher K., Reimer R.A., Millet G., Fung T., Culos-Reed S. (2022). Effects of a 12-Week HIIT + Group Mediated Cognitive Behavioural Intervention on Quality of Life among Inactive Adults with Coeliac Disease: Findings from the Pilot MOVE-C Study. Psychol. Health.

[69] Wolf R.L., Morawetz M., Lee A.R., Koch P.A., Contento I.R., Zybert P., Green P.H.R., Lebwohl B. (2020). A Cooking-Based Intervention Promotes Gluten-Free Diet Adherence and Quality of Life for Adults with Celiac Disease. Clin. Gastroenterol. Hepatol..

[70] Haas K., Martin A., Park K.T. (2017). Text Message Intervention (TEACH) Improves Quality of Life and Patient Activation in Celiac Disease: A Randomized Clinical Trial. J. Pediatr..

[71] Sainsbury K., Mullan B., Sharpe L. (2013). A Randomized Controlled Trial of an Online Intervention to Improve Gluten-Free Diet Adherence in Celiac Disease. Am. J. Gastroenterol..

[72] van der Graaf J. (2020). Inquiry-Based Learning and Conceptual Change in Balance Beam Understanding. Front. Psychol..

[73] Ali A. (2014). The Effect of Inquiry-Based Learning Method on Students’ Academic Achievement in Science Course. Univers. J. Educ. Res..

[74] Duran M., Dökme İ. (2016). The Effect of the Inquiry-Based Learning Approach on Student’s Critical-Thinking Skills. Eurasia J. Math. Sci. Technol. Educ..

[75] Alkhalifa F.M., Abu Deeb F.A., Al-Saleh W.M., Al Hamad S.S., Adams C. (2023). Knowledge of and Behaviors toward a Gluten-Free Diet among Women at a Health Sciences University. J. Taibah Univ. Med. Sci..

[76] Russo C., Wolf R.L., Leichter H.J., Lee A.R., Reilly N.R., Zybert P., Green P.H.R., Lebwohl B. (2020). Impact of a Child’s Celiac Disease Diagnosis and Management on the Family. Dig. Dis. Sci..

[77] Oflu A.T., Bukulmez A., Icigen E., Molon L., Koca T.G., Avsar Y.E. (2020). Life Quality and Empathy in Children with Celiac Disease. North. Clin. Istanb..

[78] Tikly L., Vogel E., Kurvers C. (2020). ERIC-ED612225—Boosting Gender Equality in Science and Technology: A Challenge for TVET Programmes and Careers.

[79] Lawrence D.A., Mancuso T.A. (2012). Promoting Girls’ Awareness and Interest in Engineering. Technol. Eng. Teach..

[80] Dasgupta N., Stout J.G. (2014). Girls and Women in Science, Technology, Engineering, and Mathematics. Policy Insights Behav. Brain Sci..

[81] Master A., Cheryan S., Moscatelli A., Meltzoff A.N. (2017). Programming Experience Promotes Higher STEM Motivation among First-Grade Girls. J. Exp. Chil. Psychol..

[82] Aeschlimann B., Herzog W., Makarova E. (2016). How to Foster Students’ Motivation in Mathematics and Science Classes and Promote Students’ STEM Career Choice. A Study in Swiss High Schools. Int. J. Educ. Res..

[83] Pablos A., Nebot V., Vañó-Vicent V., Ceca D., Elvira L. (2018). Effectiveness of a School-Based Program Focusing on Diet and Health Habits Taught through Physical Exercise. Appl. Physiol. Nutr. Metab..

[84] Santos-Beneit G., Bodega P., de Miguel M., Rodríguez C., Carral V., Orrit X., Haro D., Carvajal I., de Cos-Gandoy A., Peñalvo J.L. (2019). Rationale and Design of the SI! Program for Health Promotion in Elementary Students Aged 6 to 11 Years: A Cluster Randomized Trial. Am. Heart J..

[85] Adab P., Barrett T., Bhopal R., Cade J.E., Canaway A., Cheng K.K., Clarke J., Daley A., Deeks J., Duda J. (2018). The West Midlands ActiVe Lifestyle and Healthy Eating in School Children (WAVES) Study: A Cluster Randomised Controlled Trial Testing the Clinical Effectiveness and Cost-Effectiveness of a Multifaceted Obesity Prevention Intervention Programme Targeted at Children Aged 6-7 Years. Health Technol. Assess..

[86] Gatto N.M., Martinez L.C., Spruijt-Metz D., Davis J.N. (2017). LA Sprouts Randomized Controlled Nutrition, Cooking and Gardening Programme Reduces Obesity and Metabolic Risk in Hispanic/Latino Youth. Pediatr. Obes..

[87] Scherr R.E., Linnell J.D., Smith M.H., Briggs M., Bergman J., Brian K.M., Dharmar M., Feenstra G., Hillhouse C., Keen C.L. (2014). The Shaping Healthy Choices Program: Design and Implementation Methodologies for a Multicomponent, School-Based Nutrition Education Intervention. J. Nutr. Educ. Behav..

[88] Scherr R.E., Linnell J.D., Dharmar M., Beccarelli L.M., Bergman J.J., Briggs M., Brian K.M., Feenstra G., Hillhouse J.C., Keen C.L. (2017). A Multicomponent, School-Based Intervention, the Shaping Healthy Choices Program, Improves Nutrition-Related Outcomes. J. Nutr. Educ. Behav..

