# Supplementary material for: Assessment of a Non-Randomized Education Intervention for Primary School Aimed to Promote the Inclusion of People with Celiac Disease: Zeliakide Project (Part II)
Source: Nutrients. 2026 Jun 3;18(11):1798. doi: 10.3390/nu18111798 (PMC13259535; doi:10.3390/nu18111798)
Supplement: Supplementary file 1 [file nutrients-18-01798-s001.zip › SUPPLEMENTARY file 1 proofs.pdf]

SUPPLEMENTARY MATERIAL 1. KEY ASPECTS OF ZELIAKIDE PROGRAM

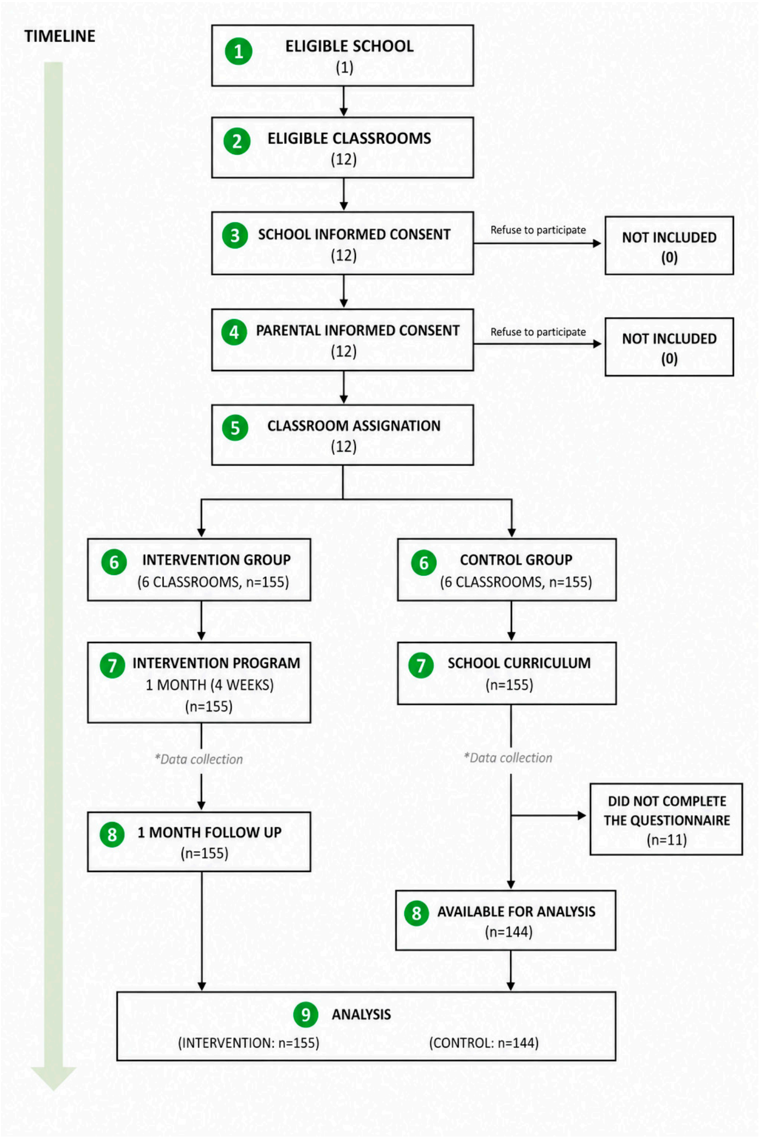

Figure S1. Study design and intervention schema of Zeliakide program.

Table S1.1. Methodological design of the ZELIAKIDE intervention [61]

Domain	Description
**Study design**	Two-armed cluster, non-randomised controlled trial
**Setting**	Primary school (Spain)
**Participants**	Children aged 10–12 years (5th–6th grade)
**Needed sample size**	144 participants
**Group allocation**	Convenience-based; intervention (5th grade) vs control (6th grade within same schools)

**Intervention duration**	4 weeks
**Session structure**	8 face-to-face sessions (2/week; 1 hour/session)
**Educational approach**	STEAM framework using Inquiry-Based Learning (IBL)
**Intervention focus**	1. Balanced diet 2. Celiac disease (CD) and gluten 3. Social inclusion of CD patients
**Pedagogical design**	Competency-based, aligned with Bloom's taxonomy (levels 1–6)
**Teaching strategies**	Games, experiments, case studies, group work, home-based activities
**Materials**	Workbook, digital classroom, experimental kits
**Family involvement**	Home assignments reinforcing session content
**Outcome assessment timing**	Pre-intervention, post-intervention, and 1-month follow-up
**Primary outcomes**	Changes in knowledge (nutrition, CD, gluten) and behaviours (diet quality, social attitudes)
**Secondary outcomes**	Parent-reported perceptions and child satisfaction
**Data collection method**	Structured questionnaires administered in sessions
**Statistical approach**	Descriptive analysis. Tests for non-parametric repeated-measures. Mann-Whitney U test to compare subgroups.

Table S.1.2. Intervention content and timeline [61]

Week	Session	Topic	Core Activities
**Week 1**	Day 1	Food groups	Food pyramid game
	Day 2	Healthy diet	Dietary recall + snack challenge introduction
**Week 2**	Day 3	Celiac disease	Disease understanding game
	Day 4	What gluten is	Hands-on experiments with dough
**Week 3**	Day 5	Gluten-containing vs gluten-free foods	Sensory analysis
	Day 6	Food labelling	Label-reading exercises
**Week 4**	Day 7	Gluten sources	Gluten detection experiment
	Day 8	Social inclusion	Case study & discussion

Cross-cutting component: Healthy snacks challenge (daily, group-based behavioural intervention).

Table S1.3. Competences and Learning Outcomes of the Zeliakide program and its corresponding level of abstraction according to Bloom [61-63].

	Competence	Learning Outcomes	Study Results
**C1**	The student should be able to place the food groups in the food pyramid.	*Bloom level 3. Application*<br>The student adequately organises different foods in the pyramid.	
**C2**	The student should be able to identify dietary errors and make recommendations for its modification.	*Bloom level 4. Analysis*<br>The student identifies foods to be consumed infrequently.<br>*Bloom level 6. Evaluation*<br>The student offers recommendations to adapt its consumption.	[60]
**C3**	The student should be able to consume less food from the top of the pyramid.	*Bloom level 6. Evaluation*<br>Students consume less unhealthy food than before the activities.	
**C4**	The student should be able to explain what celiac disease is.	*Bloom level 2. Understanding*<br>The student acknowledges that gluten harms people with celiac disease and makes them sick.<br>The student concludes that people with CD should follow a gluten-free diet.	
**C5**	The student should be able to analyse gluten	*Bloom level 3. Application*<br>The student manipulates gluten and finds it.<br>The student figures out that gluten gives elasticity to the doughs.<br>*Bloom level 4. Analysis*<br>Students deduce where gluten is by analysing food labels.	
**C6**	The student should be able to classify food groups according to gluten content.	*Bloom level 4. Analysis*<br>The student identifies the food groups that contain gluten: placing them in cereals.<br>The student concludes that processed foods may contain gluten.	Present article
**C7**	The student should be able to understand the work of scientists, to know that the gluten content of foods is analysed through experimentation.	*Bloom level 6. Evaluation*<br>The student is able to perform experimental work, observe, analyse and evaluate the results.	
**C8**	The student should be able to assess the impact of own actions on others.	*Bloom level 6. Evaluation*<br>The student demonstrates empathetic behaviour.<br>The student selects actions and behaviours to overcome differences between people.	
