# Supplementary material for: Assessment of a Non-Randomized Education Intervention for Primary School Aimed to Promote the Inclusion of People with Celiac Disease: Zeliakide Project (Part II)
Source: Nutrients. 2026 Jun 3;18(11):1798. doi: 10.3390/nu18111798 (PMC13259535; doi:10.3390/nu18111798)
Supplement: Supplementary file 1 [file nutrients-18-01798-s001.zip › SUPPLEMENTARY file 2.pdf]

SUPPLEMENTARY MATERIAL 2. Questionnaire to evaluate the programme.

Competence	Question	Response
C4	How much do you know about CD?	Scale 0 (nothing) – 4 (much)
	What symptoms do people with CD have?	Open question
	When do people with CD develop symptoms?	Open question
	What compound in food is harmful to people with CD?	Open question
C5	How do you think people with CD feel?	Open question
	Does a person with CD enjoy eating?	Scale 0 (nothing) – 4 (much)
	What would you do if it was your birthday and a classmate had CD?	Bring the cake that I like the most<br><br>Bring the cake that I like the most and another gluten-free cake for the person with CD<br><br>Bring a gluten-free cake for everyone<br><br>No response
C6	Where is the gluten?	Open question
	What makes a dough elastic?	The gluten/The water/I do not know
C7	Which of the following foods a person with CD cannot eat?	Meat and fish/eggs and dairy products/fruits and vegetables/products made with wheat (biscuits, bread, pasta)/rice and corn/sweets/they can eat everything/I do not know
	From the following list of foods, which ones may contain gluten?	Raw rice/Raw beans/fresh fruit/fresh vegetables/natural dairy products/fresh meat and fish/biscuits/some sweets/sweetened cocoa powder/some ice-creams/chocolate
	Can a prepared food (cream of vegetables, prepared beans...) have gluten, even if it is made from gluten-free ingredients?	Yes, it can be added during preparation.<br><br>No, if the ingredients are gluten-free, it is impossible for the dish to contain gluten.
	How can we know (without experimenting) whether a food contains gluten or not?	Open question
	Which symbols indicate that a food does not contain gluten?	Open question

C8	Rate these sentences according to your degree of agreement:<br>In order to detect gluten, laboratory experiments must be carried out.<br>It is important for scientists to do experiments.<br>In the future, I would like to become a scientist.<br>Researchers are crazy, weird and male.	Scale 1 (not agree) – 5 (very agree)
