# Supplementary material for: Assessment of a Non-Randomized Education Intervention for Primary School Aimed to Promote the Inclusion of People with Celiac Disease: Zeliakide Project (Part II)
Source: Nutrients. 2026 Jun 3;18(11):1798. doi: 10.3390/nu18111798 (PMC13259535; doi:10.3390/nu18111798)
Supplement: Supplementary file 1 [file nutrients-18-01798-s001.zip › SUPPLEMENTARY file 3.pdf]

## SUPPLEMENTARY MATERIAL 3. DETAILED RESULTS TABLES.

**Table S3.1.** Questions and answers to evaluate the competence 6: analysing gluten.

| Question | Type of response | Pre response Quantity (%) or Mean ± SD | Post response Quantity (%) or Mean ± SD | *p* |
|---|---|---|---|---|
| Where is the gluten? *(open question)* | Scale between 0 (do not know the answer) – 4 (do know the answer) | 1.79 ± 1.79 | 2.91 ± 1.52 | <0.001 |
| What makes a dough elastic? | He/She/They know the answer | - | 123 (79.4%) | - |
| | He/She/They do not know the answer | - | 25 (16.1%) | - |
| | No response | - | 7( 4.5%) | - |

SD: standard deviation

**Table S3.2.** Evaluation of the competence 8: degree of agreement with the statements regarding the work of scientists.

| Statement | Type of response | Pre response Mean ± SD | Post response Mean ± SD | *p* |
|---|---|---|---|---|
| In order to detect gluten, laboratory experiments must be carried out. | Scale 1 (not agree) – 5 (very agree) | 3.90 ± 0.92 | 4.36 ± 0.82 | <0.001 |
| It is important for scientists to do experiments. | | 4.23 ± 0.84 | 4.35 ± 0.74 | 0.065 |
| In the future, I would like to become a scientist. | | 3.46 ± 1.02 | 3.63 ± 1.16 | <0.05 |
| Researchers are crazy, weird and male. | | 2.95 ± 1.30 | 2.83 ± 1.37 | NS |

NS: non-significant; SD: standard deviation

**Table S3.3**. Control group results comparison with intervention group.

| Question | Type of response | Pre response Quantity (%) or Mean ± SD | Post response Quantity (%) or Mean ± SD | RT response Quantity (%) or Mean ± SD | Control group response Quantity (%) or Mean ± SD | *p (Pre and control group)* | *p (Post and control group)* | *p (RT and control group)* |
|---|---|---|---|---|---|---|---|---|
| How much do you know about CD? | Scale 0 (nothing) – 4 (much) | 1.99 ± 1.17 | 2.85 ± 1.06 | 3.12 ± 0.78 | 2.14 ± 1.10 | NS | <0.001 | <0.001 |
| What symptoms do people with CD have? *(open question)* | He/She/They know the answer | 15 (9.7%) | 93 (60%) | 106 (68.4%) | 22 (15.3%) | NS | <0.001 | <0.001 |
| | He/She/They do not know the answer | 133 (85.8%) | 51 (32.9%) | 40 (25.8%) | 122 (84.7%) | | | |
| | No response | 7 (4.5%) | 11 (7.1%) | 9 (5.8%) | 0 (0%) | | | |
| What compound in food is harmful to people with CD? *(open question)* | He/She/They know the answer | 33 (21.3%) | 82 (52.9%) | 86 (55.5%) | 87 (60.4%) | <0.001 | NS | NS |
| | He/She/They do not know the answer | 114 (73.5%) | 63 (40.6%) | 59 (38.1%) | 57 (39.6%) | | | |
| | No response | 8 (5.2%) | 10 (6.5%) | 10 (6.5%) | 0 (0%) | | | |
| What would you do if it was your | Bring the cake that I like the most. | 6 (3.9%) | 4 (2.6%) | 9 (5.8%) | 8 (5.6%) | <0.05 | <0.001 | <0.001 |

| | | | | | | | | |
|---|---|---|---|---|---|---|---|---|
| birthday and a classmate had CD? | Bring the cake that I like the most and another gluten-free cake for the person with CD. | 62 (40%) | 21 (13.5%) | 32 (20.6%) | 81 (56.3%) | | | |
| | Bring a gluten-free cake for everyone. | 82 (52.9%) | 124 (80%) | 105 (67.7%) | 55 (38.2%) | | | |
| | No response | 5 (3.2%) | 6 (3.9%) | 9 (5.8%) | 0 (0%) | | | |
| Where is the gluten? *(open question)* | Scale between 0 (do not know the answer) – 4 (do know the answer) | 1.79 ± 1.79 | 2.91 ± 1.52 | 2.48 ± 1.67 | 1.61 ± 1.82 | NS | <0.001 | <0.001 |
| Which of the following food groups can a person with CD not eat? | He/She/They know the answer | 121 (78.1%) | 125 (80.6%) | 121 (78.1%) | 101 (70.1%) | <0.05 | <0.01 | <0.05 |
| | He/She/They do not know the answer | 25 (16.1%) | 21 (13.5%) | 25 (16.1%) | 43 (29.9%) | | | |
| | No response | 9 (5.8%) | 9 (5.8%) | 9 (5.8%) | 0 (0%) | | | |
| Can a prepared food (cream of vegetables, prepared beans...) have gluten, even if it is made from gluten-free ingredients? | He/She/They know the answer | 102 (65.8%) | 122 (78.7%) | 113 (72.9%) | 102 (70.8%) | NS | <0.05 | NS |
| | He/She/They do not know the answer | 45 (29%) | 26 (16.8%) | 33 (21.3%) | 42 (29.2%) | | | |
| | No response | 8 (5.2%) | 7 (4.5%) | 9 (5.8%) | 0 (0%) | | | |
| How can we know (without experimenting) whether a food contains gluten or not? *(open question)* | Scale between 0 (do not know the answer) – 3 (do know the answer) | 0.59 ± 1.09 | 2.21 ± 1.30 | 2.07 ± 1.33 | 1.06 ± 1.42 | <0.01 | <0.001 | <0.001 |
| In order to detect gluten, laboratory experiments must be carried out. | Scale 1 (not agree) – 5 (very agree) | 3.90 ± 0.92 | 4.36 ± 0.82 | - | 3.01 ± 1.07 | <0.001 | <0.001 | - |
| It is important for scientists to do experiments. | Scale 1 (not agree) – 5 (very agree) | 4.23 ± 0.84 | 4.35 ± 0.74 | - | 3.94 ± 0.91 | <0.01 | <0.001 | |

CD: celiac disease; NS: non-significant

**Table S3.4.** Questions related to the interest shown by the children according to the parents (n=108).

| Question | Response | Frequency of response (%) or mean ± SD |
|---|---|---|
| Has the child talked at home about what he/she/they has done with a group of researchers? | Yes | 103 (95.4%) |
| | No | 5 (4.6%) |
| If he/she has commented, what would you say his/her/their interest has been? | Scale 1 (none) -10 (much) | 7.94 ± 1.79 |

| | | |
|---|---|---|
| Has the child taught you something about the subject that you did not know? | Yes<br>No | 76 (70.4%)<br>32 (29.6%) |
| Has the child done the following homework? | | |
| Write down everything you have eaten during the day* | Yes<br>No<br>I do not know | 101 (93.5%)<br>5 (4.6%)<br>2 (1.9%) |
| Analyse food advertisements in the media* | Yes<br>No<br>I do not know | 89 (82.4%)<br>15 (13.9%)<br>4 (3.7%) |
| Putting yourself in the shoes of a person with CD | Yes<br>No<br>I do not know | 67 (62.0%)<br>33 (30.6%)<br>8 (7.4%) |
| Prepare and sensorially analyse gluten and gluten-free pasta | Yes<br>No<br>I do not know | 63 (58.3%)<br>45 (41.7%)<br>0 (0%) |
| Analyse food labelling and consider what people with CDs should do in the case of unpackaged food | Yes<br>No<br>I do not know | 89 (83.2%)<br>17 (15.9%)<br>(0.9%) |

*These homework assignments were from part 1 of the programme [56]. CD: celiac disease; SD: standard deviation

**Table S3.5.** Questions and answers in relation to parents' opinions (n=108).

| Question | Response | Frequency of response (%) or mean ± SD |
|---|---|---|
| Before the Zeliakide programme, did you know about CD and GFD? | Scale 1 (I did not know about it) – 4 (I knew it in depth) | 2.67 ± 0.79 |
| If the kid has not done his or her homework, can you indicate the reason? *(Open question)* | She/He/They has done all the homework<br>Lack of time<br>Lack of interest or they decided not to do it. | 49 (52.1%)<br>26 (27.7%)<br>19 (20.2%) |
| How interesting did you find the Zeliakide programme? | Scale 1 (none) -10 (much) | 8.37 ± 1.47 |
| Would you recommend this programme to other schools? | Yes<br>No<br>Perhaps<br>I do not know | 101 (93.5%)<br>3 (2.8%)<br>3 (2.8%)<br>1 (0.9%) |

CD: celiac disease; GFD: gluten-free diet; SD: standard deviation
